# Bistatic Radar Cooperative Imaging Based on Complementary Random Waveform

**DOI:** 10.3390/s23052577

**Published:** 2023-02-25

**Authors:** Xin Li, Jiemin Hu, Bo Zou, Yongfeng Zhu, Zhiyong Song

**Affiliations:** 1 School of Information Science and Engineering, Zhejiang Sci-Tech University, Hangzhou 310018, China; 2 School of Computer Science and Technology, Zhejiang Sci-Tech University, Hangzhou 310018, China; 3 Institute of Land Aviation, Beijing 101121, China; 4 College of Electronic Science and Engineering, National University of Defense Technology, Changsha 410073, China

**Keywords:** bistatic radar, random frequency-hopping, cooperative imaging, motion compensation

## Abstract

The cooperative imaging of the bistatic radar is an important research topic for missile-borne radar detection. The existing missile-borne radar detection system is mainly based on the target plot information separately extracted by each radar for data level fusion, without considering the gain brought by the cooperative processing of the radar target echo signal. In this paper, a random frequency-hopping waveform is designed for the bistatic radar to achieve efficient motion compensation. A coherent processing algorithm for bistatic echo signals is designed to achieve band fusion and improve the signal quality and range resolution of the radar. Simulation and high-frequency electromagnetic calculation data were used to verify the effectiveness of the proposed method.

## 1. Introduction

Radars with all-weather, all-day, long-range detection ability, are one of the important sensors in the onboard platforms for target imaging [1]. However, with the progress of target stealth technology and the rapid technical development of jamming countermeasures, such as target RCS reduction, which requires a radar to adopt a large antenna aperture and signal system with a large bandwidth, which brings great challenges to single radar detection [2]. Compared to single radar detection, multi-radar collaborative detection can not only obtain more observation periods but also obtain the characteristics of different dimensions, such as frequency character [3], polarization character [4], space character [5], etc., which is an important development direction for stealth target detection in a complex electromagnetic environment. According to the varied detection angles and radar working frequency bands, multi-radar cooperative detection mode can be divided into spatial cooperation and frequency band cooperation, to obtain the benefits of spatial diversity [6,7] and frequency diversity [8,9]. The former realizes target detection and recognition by jointly processing information for multiple detection angles to reduce the probability of misjudgment in single-view detection. The latter processes varied radar frequencies coherently to obtain higher-range resolution and gain by pulse compression.

For the application scenario of the missile-borne radar, multiple missile-borne radars accompany the flight, and the difference in detection perspective is small. Frequency band coordination is usually adopted to improve detection performance. Moreover, the missile-borne radar usually works in complex electromagnetic environments with a large number of jammers. In order to improve the detection performance, the missile-borne radar usually adopts a random frequency-hopping signal with excellent anti-jamming performance [10,11,12,13]. Therefore, based on the bistatic radar detection framework and the random frequency-hopping signal system, this paper studies the bistatic radar cooperative imaging method. Due to the movement of different radars with high speed and the distribution of echo data in discontinuous multiple frequency bands, it is necessary to eliminate the phase error introduced by the platform motion and compensate for the phase difference between the varied radars. Furthermore, the data fusion of different frequency bands needs to be realized. The process involves motion compensation, coherent registration, and data fusion [14,15,16]. In terms of motion compensation, reference [17] used the cross-correlation of adjacent range directions for velocity estimation. The compensation accuracy is determined by the bandwidth, which cannot be applied to radar waveforms sensitive to motion, such as random frequency-hopping. In [18], the extremum of the searching cost function determined the target velocity. However, the performance of the algorithm is sensitive to the step size and range of the searching step. In [19], a velocity estimation method based on the complementary code modulation was proposed to achieve precise velocity estimation by transmitting adjacent pulse trains with complementary relations. However, the radar data rate is reduced as two pulse trains are needed. In terms of coherent registration, [20] divided the compensated phase between subbands into a linear phase and a fixed phase and used the all-pole model to solve the problem. However, the pole order is difficult to determine. In [21], a cross-correlation method was used to solve the linear phase term, and FFT transformation was used to solve the fixed phase term, which avoided the pole order problem. In terms of frequency band data fusion, in [22,23], the defective frequency band was filled in a wideband signal for imaging. However, the band estimation was based on the existing frequency band without additional information. In [24], a probabilistic model was established for sparse-frequency band echo signals. After Doppler focusing on the target, a Bayesian learning algorithm was used for range fusion imaging to avoid the errors introduced by the frequency band filling algorithm. However, Doppler focusing requires accurate target motion compensation, and the compensation accuracy of the sub-frequency band needs further analysis.

In this paper, we studied the bistatic radar cooperative imaging based on a complementary random waveform. Firstly, we constructed a dual-platform cooperative detection geometric model of bi-transmitting and bi-receiving bistatic radars. Secondly, a random frequency-hopping signal waveform pair with complementary characteristics is proposed and analyzed. Then, the motion compensation of the target is carried out to realize the phase-coherent registration of the echo signal. Through the complementary characteristics of frequency-hopping coefficients, the distance and velocity terms of the target in the phase of the echo signal are separated, and then the target velocity is accurately estimated. Finally, a band fusion algorithm is designed to achieve two-dimensional imaging of the target. The radar imaging method studied in this paper has the advantages of the bistatic radar system and a random frequency-hopping signal [3,4,5,10,11,12,13]. It improves the detection performance and realizes the efficient estimation of target velocity, which lays a foundation for the future study of missile-borne radar collaborative detection imaging.

This paper is organized as follows. In Section 2, the waveform pairs of the bistatic random frequency-hopping signal with complementary characteristics are designed. A model of a bistatic radar echo is derived. In Section 3, the compensation phase between echoes is accurately calculated using the echoes of the homologous transmitted signals. The coherent registrations of echoes are effectively realized. Furthermore, an efficient velocity estimation method is designed based on the complementary characteristics of echoes to fulfill the relative motion compensation. Section 4 provides that the band fusion algorithm is based on tight constraint rearrangement and zero complement for bistatic radar cooperative imaging. The point-target simulation data and the high-frequency electromagnetic calculation data are used to verify the algorithms in Section 5. Finally, the conclusions are summarized in Section 6.

## 2. Echo Model of Bistatic Radar Cooperative Imaging

**Figure 1:**
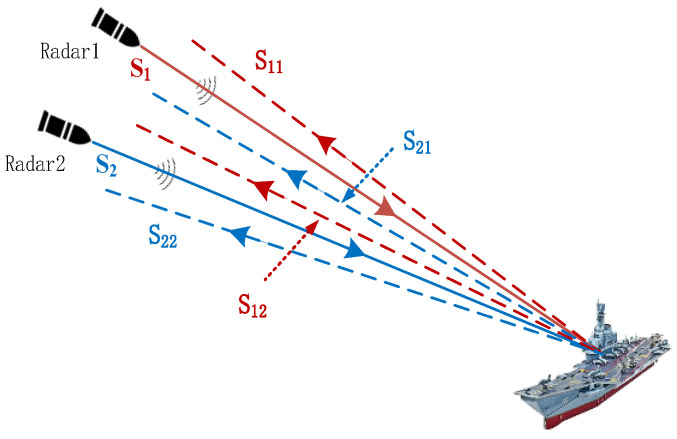
Bistatic radar collaborative imaging.

The bistatic radar cooperative imaging is shown in Figure 1. Suppose that both radars 1 and 2 adopt random frequency-hopping waveforms, and both coherent processing pulse trains contain N sub-pulses with the same frequency-hopping rules. The pulse repetition time (PRT) is $T_{r}$. The pulse width is T. The random modulation frequency distribution is within a given bandwidth B. The minimum frequency-hopping (FH) step is Δf=B/N. The FH coefficient is a random integer sequence of length N in [0,N−1]. In order to make full use of the frequency band, the frequency-hopping coefficient usually traverses the interval and is not repeated. Suppose that the FH coefficients of radars 1 and 2 are $c_{1}(n)$ and $c_{2}(n)$, respectively. The transmitted signal models of the two radars are expressed as follows:

(1) $$s_{1}(t)=\sum_{n=0}^{N-1}\mathrm{rect}\left(\frac{t-nT_{r}}{T}\right)\exp\left(j2\pi \left(f_{1}+c_{1}(n)\Delta f\right)\left(t-nT_{r}\right)\right)$$

(2) $$s_{2}(t)=\sum_{n=0}^{N-1}\mathrm{rect}\left(\frac{t-nT_{r}}{T}\right)\exp\left(j2\pi \left(f_{2}+c_{2}(n)\Delta f\right)\left(t-nT_{r}\right)\right)$$

where $f_{1}$ and $f_{2}$ are the carrier frequencies of radar 1 and 2, respectively.

In order to separate the varied radar signal components by the corresponding band pass filter, the carrier frequency difference of $f_{1}$ and $f_{2}$ for both radars should be greater than the radar signal modulation bandwidth. In Figure 1, the separated set of echoes is denoted as $\left\{s_{11}(t),s_{12}(t),s_{21}(t),s_{22}(t)\right\}$, where $s_{pq}$ represents the pulse transmitted by radar p (p=1,2) and the echo in radar q (q=1,2) receiving mode. Suppose that the target contains K scattering centers. At the initial time, the distance between the kth scattering center and radar 1 is $r_{1k}$. The radial velocity of the target is v. When the nth sub-pulse is emitted, the distance between the scattering center and radar 1 can be expressed as $r_{1k}(n)=r_{1k}+vnT_{r}$. When radar 1 transmits and radar 1 receives, the nth sub-pulse echo can be expressed as

(3) $$\begin{array}{l} s_{11}\left(n\right)=\sum_{k=0}^{K-1}\sigma_{k}\exp\left[-j\frac{4\pi }{c}(f_{1}+c_{1}(n)\Delta f)r_{1k}\left(n\right)\right]\\ \begin{array}{ccc} & & = \end{array}\sum_{k=0}^{K-1}\sigma_{k}\exp\left[-j\frac{4\pi }{c}(f_{1}+c_{1}(n)\Delta f)\left(r_{1k}+vnT_{r}\right)\right] \end{array}$$

where the $\sigma_{k}$ is the intensity of the kth scattering center. For the scene of collaborative imaging of multiple missile-borne radars, the lines of sight from varied radars are almost the same. In other words, the intensity and velocity of a scattering center can be regarded as constant for the two radars.

Suppose that, at the initial time, the distance between the kth scattering center and radar 2 is $r_{2k}$. When radar 2 transmits and radar 2 receives, the nth sub-pulse echo can be expressed as

(4) $$\begin{array}{l} s_{22}\left(n\right)=\sum_{k=0}^{K-1}\sigma_{k}\exp\left[-j\frac{4\pi }{c}(f_{2}+c_{2}(n)\Delta f)r_{2k}\left(n\right)\right]\\ \begin{array}{ccc} & & = \end{array}\sum_{k=0}^{K-1}\sigma_{k}\exp\left[-j\frac{4\pi }{c}(f_{2}+c_{2}(n)\Delta f)\left(r_{1k}+vnT_{r}+\Delta r\right)\right] \end{array}$$

where $\Delta r=r_{2k}-r_{1k}$ represents the electromagnetic wave transmission time corresponding to the distance difference between the two radars.

Similarly, the echo $s_{21}\left(n\right)$ received by radar 1 transmitted by radar 2 and echo $s_{12}\left(n\right)$ received by radar 2 transmitted by radar 1 are, respectively, expressed as

(5) $$\begin{array}{l} s_{21}\left(n\right)=\sum_{k=0}^{K-1}\sigma_{k}\exp\left[-j\frac{4\pi }{c}(f_{2}+c_{2}(n)\Delta f)\left(\frac{r_{2k}+r_{1k}}{2}+vnT_{r}\right)\right]\\ \begin{array}{ccc} & & = \end{array}\sum_{k=0}^{K-1}\sigma_{k}\exp\left[-j\frac{4\pi }{c}(f_{2}+c_{2}(n)\Delta f)\left(r_{1k}+vnT_{r}+\frac{\Delta r}{2}\right)\right] \end{array}$$

(6) $$s_{12}\left(n\right)=\sum_{k=0}^{K-1}\sigma_{k}\exp\left[-j\frac{4\pi }{c}(f_{1}+c_{1}(n)\Delta f)\left(r_{1k}+vnT_{r}+\frac{\Delta r}{2}\right)\right]$$

## 3. Motion Compensation Method Based on Complementary Random Waveform

### 3.1. Distance Difference Compensation

Comparing Equation (3) with Equation (6), the echo signals transmitted by one radar and received by another radar have phase modulation terms introduced by different distance delay. $s_{12}\left(n\right)$ can be represented by $s_{11}\left(n\right)$ as

(7) $$\begin{array}{l} s_{12}\left(n\right)=\exp\left[-j\frac{2\pi \Delta r}{c}(f_{1}+c_{1}(n)\Delta f)\right]\sum_{k=0}^{K-1}\sigma_{k}\exp\left[-j\frac{4\pi }{c}(f_{1}+c_{1}(n)\Delta f)\left(r_{1k}+vnT_{r}\right)\right]\\ \begin{array}{ccc} & & = \end{array}\exp\left[-j\frac{2\pi \Delta r}{c}(f_{1}+c_{1}(n)\Delta f)\right]s_{11}\left(n\right)\\ \begin{array}{ccc} & & = \end{array}\exp\left[-j\phi (n)\right]s_{11}\left(n\right) \end{array}$$

where $\phi (n)=2\pi \Delta r(f_{1}+c_{1}(n)\Delta f)/c$ and ϕ(n) can be calculated from $s_{12}\left(n\right)$ and $s_{11}\left(n\right)$, and it is used to compensate the range difference of $s_{12}(n)$. ϕ(n) can be expressed as

(8) $$\phi (n)=-angle\left\{s_{12}(n)\cdot conj\left[s_{11}(n)\right]\right\}$$

The compensation process of $s_{22}(n)$ and $s_{21}(n)$ is similar. The echo after range compensation can be expressed as

(9) $$s_{11}\left(n\right)=s_{12}\left(n\right)=\sum_{k=0}^{K-1}\sigma_{k}\exp\left[-j\frac{4\pi }{c}(f_{1}+c_{1}(n)\Delta f)\left(r_{1k}+vnT_{r}\right)\right]$$

(10) $$s_{21}\left(n\right)=s_{22}\left(n\right)=\sum_{k=0}^{K-1}\sigma_{k}\exp\left[-j\frac{4\pi }{c}(f_{2}+c_{2}(n)\Delta f)\left(r_{1k}+vnT_{r}\right)\right]$$

After the radar range difference is compensated, the echoes emitted by varied radars have the same law. Therefore, the collaborative imaging method is studied based on echoes $s_{11}\left(n\right)$ and $s_{21}\left(n\right)$.

### 3.2. Echo Motion Compensation

It can be seen from the above echo model that there is a coupling term between the frequency variation and range variation of the random frequency-hopping waveforms, which will lead to the introduction of a higher order phase term in the echo. The high-resolution range profile obtained by direct FFT has a serious defocus. It is necessary to compensate for the target motion. By comparing Equations (9) and (10), it can be seen that $s_{11}\left(n\right)$ and $s_{21}\left(n\right)$ have the same range delay and different signal frequencies. If the random frequency-hopping mode of the two radars is designed reasonably, the influence of frequency modulation can be eliminated to achieve the rapid estimation of target motion parameters.

**Figure 2:**
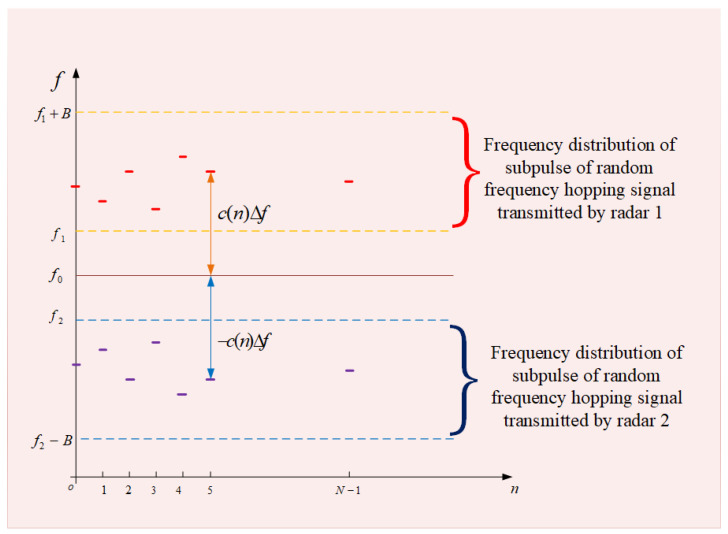
Bistatic radar random frequency-hopping complementary modulation.

The random frequency-hopping modulation of the two radars is shown in Figure 2, where $f_{0}=\left(f_{1}+f_{2}\right)/2$, $c(n)=c_{1}(n)+\left(f_{2}-f_{1}\right)/2$ and $c_{2}(n)=-c_{1}(n)$. Both random frequency-hopping signals from radars comply with the frequency rules. The frequency-hopping coefficient c(n) of the pulse transmitted by radar 1 equals to the one of radar 2, and the direction is opposite. It can be obtained by substituting the above equations into Equations (9) and (10).

(11) $$s_{11}\left(n\right)=\exp\left[-j\frac{4\pi vnT_{r}}{c}(f_{0}+c(n)\Delta f)\right]\sum_{k=0}^{K-1}\sigma_{k}\exp\left[-j\frac{4\pi }{c}(f_{0}+c(n)\Delta f)r_{1k}\right]$$

(12) $$s_{21}\left(n\right)=\exp\left[-j\frac{4\pi vnT_{r}}{c}(f_{0}-c(n)\Delta f)\right]\sum_{k=0}^{K-1}\sigma_{k}\exp\left[-j\frac{4\pi }{c}(f_{0}-c(n)\Delta f)r_{1k}\right]$$

In order to estimate the velocity of the target, the echo components $s_{11}$ and $s_{21}$ are multiplied and the following results were obtained.

(13) $$\begin{array}{ll} s\left(n\right)= & s_{11}\left(n\right)\;\;\;\cdot \;\;s_{21}\left(n\right)\\ & =\exp\left(-j\frac{8\pi }{c}vnT_{r}f_{0}\right)\sum_{k=0}^{K-1}\sum_{l=0}^{K-1}\sigma_{k}\sigma_{l}\exp\left\{-j\frac{4\pi }{c}\left[f_{0}(r_{1k}+r_{1l})+c(n)\Delta f(r_{1k}-r_{1l})\right]\right\}\\ & =\exp\left(-j\frac{8\pi }{c}vnT_{r}f_{0}\right)\left\{\sum_{k=0}^{K-1}\sigma_{k}^{2}\exp\left[-j\frac{8\pi f_{0}}{c}r_{1k}\right]\right.\\ & \left.+\sum_{k=0}^{K-1}\sum_{l=0,l\neq k}^{K-1}\sigma_{k}\sigma_{l}\exp\left[-j\frac{4\pi }{c}\left(f_{0}(r_{1k}+r_{1l})+c(n)\Delta f(r_{1k}-r_{1l})\right)\right]\right\}\\ & =\exp\left(-j\frac{8\pi }{c}vnT_{r}f_{0}\right)\left[s_{0}+s^{\prime }\left(n\right)\right] \end{array}$$

where $\sum_{k=0}^{K-1}\sigma_{k}^{2}\exp\left[-j\frac{8\pi f_{0}}{c}r_{1k}\right]$ is a constant value and $s^{\prime }\left(n\right)$ varies with the number of sub-pulses n. Therefore, $s\left(n\right)$ comprises a single-frequency signal component $s_{0}$ and a variable component $s^{\prime }\left(n\right)$. After the FFT transformation, the energy of the single-frequency signal component is coherently accumulated to form a peak, while $s^{\prime }\left(n\right)$ is still in the defocus state. As the result, the target velocity can be estimated by the peak position of the sequence after FFT transformation.

(14) $$v=\frac{k_{0}c}{4NT_{r}f_{0}}$$

where $k_{0}$ is the peak position, N=128, $T_{r}=10^{-4}s$, $f_{0}=15GHZ$. By Equation (14), the target velocity estimation precision is $\Delta v=\frac{c}{8NT_{r}f_{0}}$, with the above parameters, the target velocity estimation precision can be calculated as Δv=0.1953m/s. To ensure the focus of the range imaging, the phase introduced by the velocity error during the imaging time should be no more than π/4, namely,

(15) $$\begin{array}{cc} 2\pi \Delta vNT_{r}\frac{f_{0}}{c}\leq \frac{\pi }{4}\Rightarrow & \Delta v\leq \end{array}\frac{c}{8f_{0}NT_{r}}$$

The target velocity estimation accuracy meets the imaging requirements of the random frequency-hopping signal. After the target velocity is estimated, the target translational velocity can be compensated for Equations (11) and (12). The compensated echo can be expressed as

(16) $$\begin{array}{l} s_{11}{}^{\prime }\left(n\right)=s_{11}\left(n\right)\cdot \exp[j\frac{4\pi vnT_{r}}{c}(f_{0}+c(n)\Delta f)]\\ =\exp\left[-j\frac{4\pi vnT_{r}}{c}(f_{0}+c(n)\Delta f)\right]\cdot \sum_{k=0}^{K-1}\sigma_{k}\exp\left[-j\frac{4\pi }{c}(f_{0}+c(n)\Delta f)r_{1k}\right]\\ \cdot \exp[j\frac{4\pi vnT_{r}}{c}(f_{0}+c(n)\Delta f)]\\ =\sum_{k=0}^{K-1}\sigma_{k}\exp\left[-j\frac{4\pi }{c}(f_{0}+c(n)\Delta f)r_{1k}\right] \end{array}$$

(17) $$\begin{array}{l} s_{21}{}^{\prime }\left(n\right)=s_{21}\left(n\right)\cdot \exp\left[j\frac{4\pi vnT_{r}}{c}(f_{0}-c(n)\Delta f)\right]\\ =\exp\left[-j\frac{4\pi vnT_{r}}{c}(f_{0}-c(n)\Delta f)\right]\cdot \sum_{k=0}^{K-1}\sigma_{k}\exp\left[-j\frac{4\pi }{c}(f_{0}-c(n)\Delta f)r_{1k}\right]\\ \cdot \exp\left[j\frac{4\pi vnT_{r}}{c}(f_{0}-c(n)\Delta f)\right]\\ =\sum_{k=0}^{K-1}\sigma_{k}\exp\left[-j\frac{4\pi }{c}(f_{0}-c(n)\Delta f)r_{1k}\right] \end{array}$$

Equations (16) and (17) describe the echo of target random frequency-hopping signals in different frequency bands. In the following, the coherent fusion high-resolution imaging algorithm of random frequency-hopping echoes in different frequency bands will be studied.

## 4. Band Fusion Imaging Based on Tight Constrained Rearrangement and Zero Complement

### 4.1. Coherent Processing

The existing frequency band fusion algorithms are mainly based on two ideas. One is to use band extrapolation to fill the defect band after imaging, which is based on the existing frequency band information with no additional information. Another idea is to use a modern spectrum, sparse representation, or the Bayesian method to extract scattering center parameters for super-resolution range imaging. Because the original information of the radar data is lost in the process of parameter extraction, it is difficult to carry out two-dimensional imaging.

**Figure 3:**
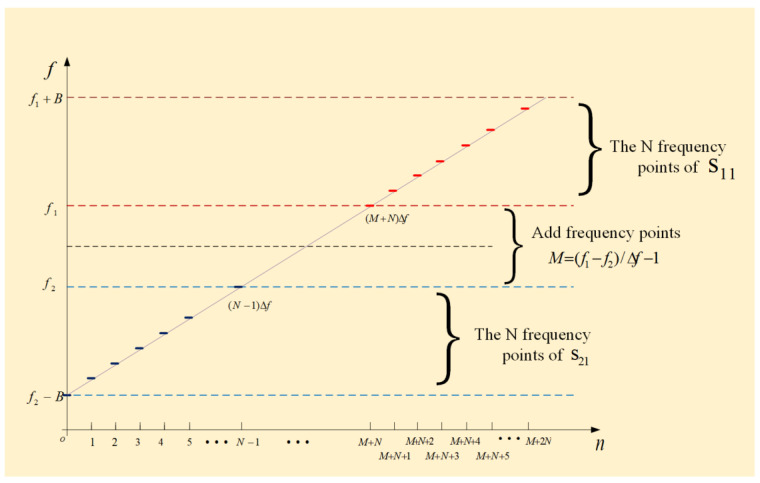
Random frequency-hopping echo rearrangement fills zero.

In this paper, a tight constrained rearrangement and zero complement frequency band fusion algorithm is proposed. The main idea is to rearrange the random frequency-hopping in different frequency bands. Zeros are made up for the echo data at the defect frequency points. Moreover, tight constraints are set so that the echo signals of the two radars can be used for coherent fusion imaging by FFT transformation. The process of zero rearrangement and complement is shown in Figure 3. The echo sequences of Equations (16) and (17) are sorted from small to large according to the minimum FH step. The obtained echo sequence is expressed as

(18) $$s_{all}\left(n\right)=\left[s_{all}\left(0\right),s_{all}\left(1\right),s_{all}\left(2\right),\ldots ,s_{all}\left(L-1\right)\right]$$

where L=M+2N is the length of echo sequence after zero padding. The first N components of $s_{all}\left(n\right)$ are the results of the $s_{21}\left(n\right)$ rearrangement. The last N components are the results of the $s_{11}\left(n\right)$ rearrangement, which are all valid observations. The defect frequency band is $\left[f_{2},f_{1}\right]$. In order to ensure the phase-coherence of the two radar FH signals, tight constraints are set: the defect bandwidth $f_{1}-f_{2}$ is an multiple of the FH step Δf, and the corresponding number of zero-padding frequency points is $M=\left(f_{1}-f_{2}\right)/\Delta f-1$.

The zero-padding position of $s_{all}\left(n\right)$ locates in the middle of defect frequency band. The range resolution of $s_{all}\left(n\right)$ is consistent with the performance of the echo sequence with continuous bandwidth 2B. The high-resolution range image obtained by FFT of $s_{all}\left(n\right)$ can be expressed as

(19) $$hrrp\left(k\right)=FFT\left[S_{all}\left(n\right)\right]$$

Thus, the range imaging results with high-resolution under bistatic cooperative imaging can be obtained. The two-dimensional range image of the target with high-resolution can be obtained by further combining the envelope alignment and the initial phase correction algorithm.

### 4.2. Imaging Process

**Figure 4:**
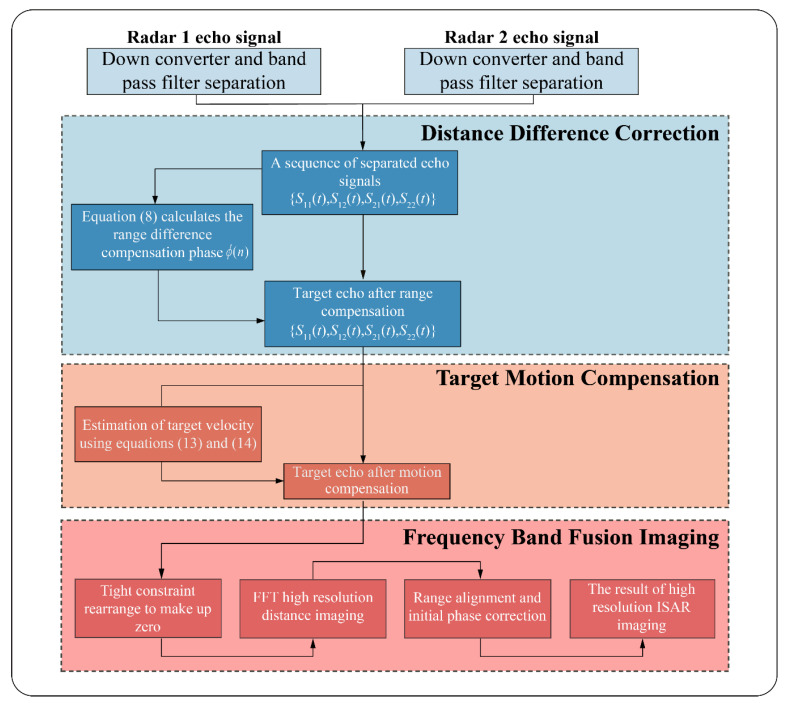
Collaborative imaging flow of the bistatic radar based on complementary random waveform.

The process of bistatic radar cooperative imaging based on complementary random waveform is shown in Figure 4. First, the echo signals of two radars that meet the complementary characteristics and tight constraints are designed. Then varied band pass filters are set according to the difference of carrier frequencies to obtain the separated target echo sequences $\left\{s_{11}(t),s_{12}(t),s_{21}(t),s_{22}(t)\right\}$.

After obtaining the echo components of the four paths, the modulation phase term introduced by the difference in distance delay is calculated under different paths according to Equation (7). It is used to compensate for the additional phase introduced by the distance difference under different paths. The distance difference correction of different paths is completed. Four-path echo signals with the same distance delay are obtained (As shown in Equations (9) and (10)).

With four-path echo signals, the same range delays are obtained. After multiplication, combined with FFT, the target speed can be effectively estimated. The target echo is compensated based on the estimated velocity, and the random frequency-hopping echo sequences of different frequency bands are obtained. Then, the range imaging of random frequency-hopping echoes in different frequency bands with high-resolution is realized by rearranging and zero padding. Finally, combined with the envelope alignment and the initial phase correction methods in the ISAR imaging process, the two-dimensional imaging of the bistatic cooperative imaging with high-resolution is completed.

The distance difference correction process requires 2M multiplication and N addition. The target motion compensation process requires 2M multiplication, N addition and one FFT. The frequency band fusion imaging requires M multiplication and 2 FFT. Thus, the complexity of the proposed imaging algorithm is o(3MN∗log(MN)+5M+2N). Where, N is the number of pulses and M is the number of sampling points.

## 5. Experimental Results

### 5.1. Validation of Simulation Data

**Figure 5:**
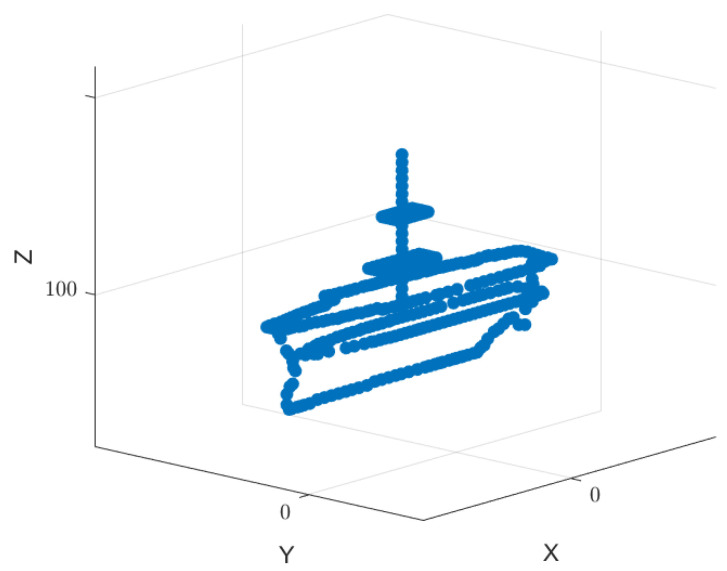
Target point scattering model.

**Figure 6:**
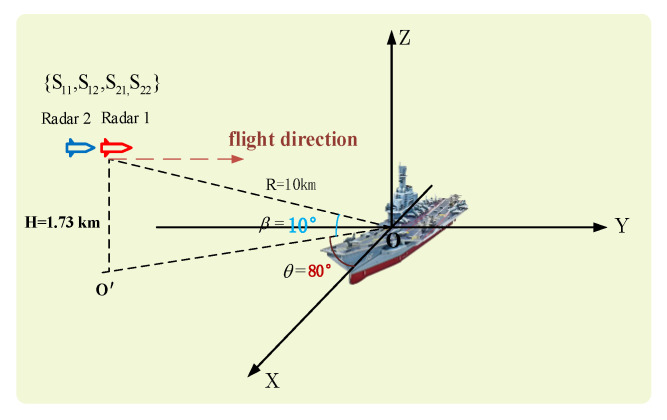
Schematic diagram of the geometric scene of dual-base cooperative imaging.

In order to verify the effectiveness of the dual base cooperative imaging method, a simulation is adopted. The point scattering model of a target ship is shown in Figure 5, which is composed of 367 scattering points. The ship size is l=120, b=30. The carrier frequency of radar 1 is $f_{1}=15\mathrm{GHz}$. The carrier frequency of radar 2 is $f_{2}=15.255\mathrm{GHz}$. The signal bandwidth is B=128MHz and Δf=1MHz. The modulated signal $c_{1}(n)$ from radar 1 is a sequence of non-repeating random integers of length N in interval $\left[0,N-1\right]$. The modulated rule from radar 2 is $c_{2}(n)=-c_{1}(n)$. The geometric scene of the bistatic cooperative imaging is shown in Figure 6, with the ship located at the origin of the coordinate system OXYZ. The bistatic imaging platform flies forward along the Y-axis at altitude H=1.73km and has the flight velocity $v_{r}=225m/s$. The distance between radar 1 and the center of the ship at the initial moment is 10 km. The target is in a maneuvering state, and the projected component of the moving speed on the radar line of sight is $v_{tLOS}=3m/s$. At the initial moment, the distance between radar 2 and the center of the ship is 10.017 km. The azimuth angle θ and pitch angle β in the target coordinate system are $\theta =80^{o}$ and $\beta =10^{o}$, respectively.

**Figure 7:**
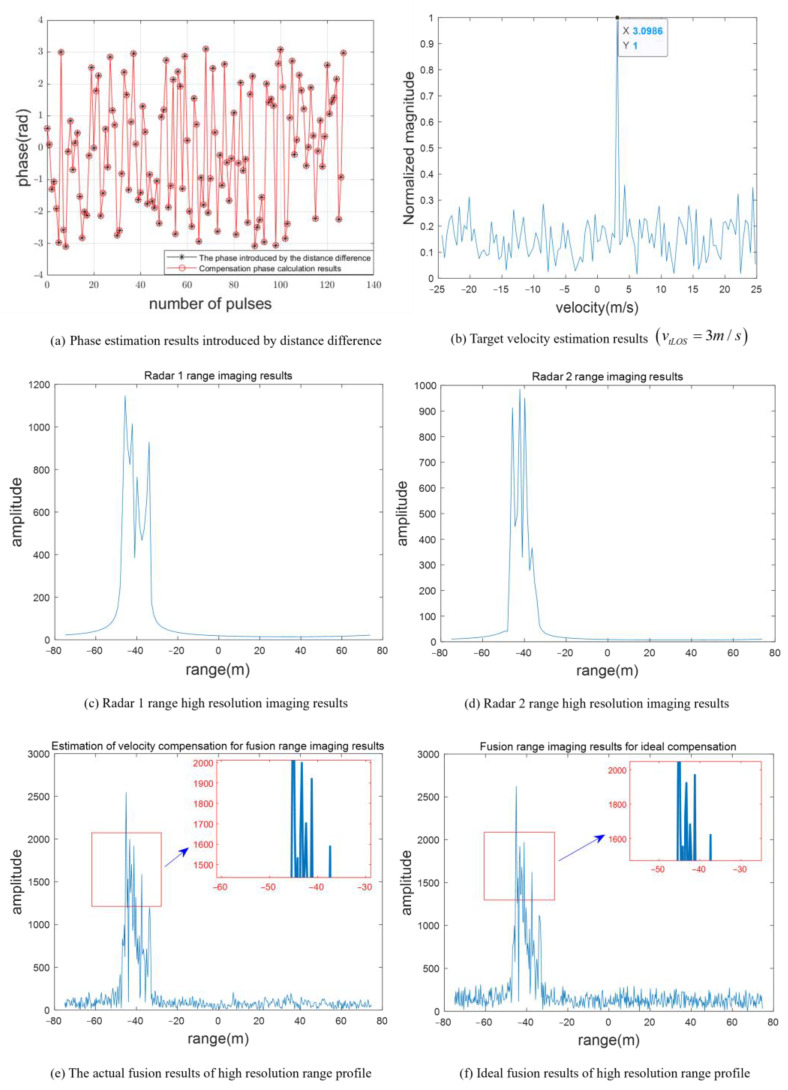
Comparison of fusion distance imaging effect.

In this paper, the complementary random frequency-hopping waveform designed is used to detect the target. The target echo sequence after separation using the band pass filter is expressed as $\left\{s_{11}(t),s_{12}(t),s_{21}(t),s_{22}(t)\right\}$. Equation (8) is used to calculate the phase introduced by radar range difference, as shown by the black asterisk in Figure 7a, and the theoretical calculation results of this phase are also given in the figure by red circles. In Figure 7a, the proposed method can effectively eliminate the phase term introduced by the platform distance difference. Figure 7b shows the FFT transformation result of $s\left(n\right)$. By Equation (14), the target velocity estimated according to the peak position is 3.099 m/s. It meets the requirements of Equation (15) Δv≤0.1953m/s. Therefore, target range imaging has good focusing performance. Figure 7c and d show the range imaging results of the two radars, respectively. Due to the limited bandwidth, the scattering centers are distributed in a small number of range units. Few target details can be obtained. The range image with high resolution fused by the proposed method is shown in Figure 7e. Meanwhile, the high-resolution range image with ideal compensation of target velocity is given in Figure 7e. In Figure 7c and e compared to, for example, the fused range image provides richer target details than the single radar range image, and the estimated speed accuracy can meet the focusing requirements of high-resolution range imaging.

**Figure 8:**
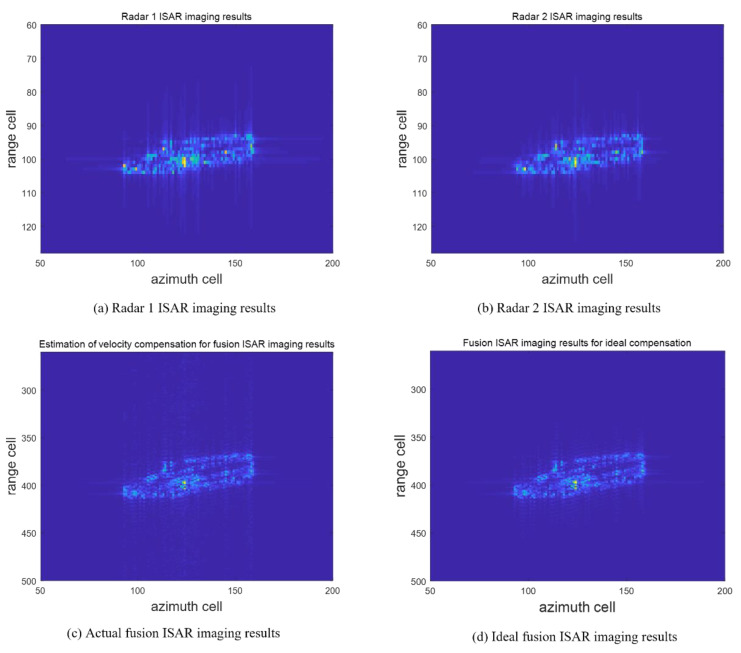
Comparison of ISAR imaging results.

**Figure 9:**
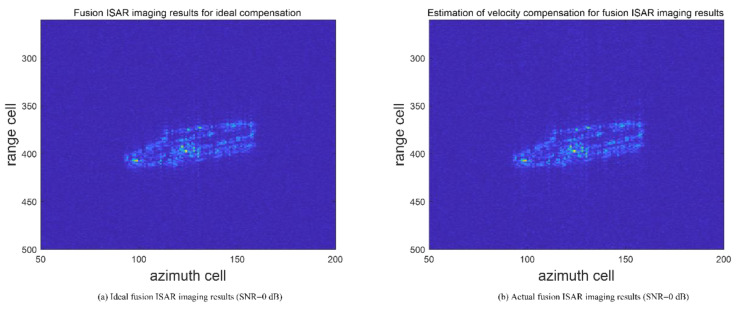
Imaging results in noisy environment.

The two-dimensional radar imaging results obtained by the envelope alignment and initial phase correction using the envelope cross-correlation method and PGA method are shown in Figure 8. Among them, Figure 8a and b show the independent imaging results of radars 1 and 2, respectively. Due to the limitation of the range resolution, the targets are distributed in about 15 range units in the range direction. The scattering center distribution is fuzzy, and the target contour is unclear. The fusion imaging results adopting the model are shown in Figure 8c. As the signal band grows, the target scattering center distribution area becomes larger, about 50 cells, providing more target details. ISAR imaging under ideal compensation of speed is presented to verify the focusing performance of the image by contrast [25]. Among them, the contrast of the actual fused ISAR image is 16.38, while the one with the ideal compensated ISAR image is 17.26. Figure 9a and b show the comparison of imaging results in a noisy environment (SNR = 0 dB). The contrast of the actual fused image in a noisy environment is 7.88. Moreover, the contrast of the ideal compensated ISAR image in a noisy environment is 8.45. Using AMD Ryzen 7 5800H with Radeon Graphics 3.20 GHz CPU and MATLAB 2020a, the average computation time for the proposed imaging algorithm is 9.18 s. The calculation time of the single base radar imaging algorithm is 8.82 s. In practical applications, the computing speed can be greatly improved by improving hardware configuration and the GPU parallel acceleration. Thus, the experimental results show that the proposed method can effectively realize the motion compensation and obtain the fusion images of the target.

### 5.2. Data Validation of High-Frequency Electromagnetic Calculations

The experimental data are the ship model shown in Figure 9. The length and width of the ship are l = 214.6 m and b = 28.04 m, respectively. The carrier frequencies of radars 1 and 2 are $f_{1}=10.1\mathrm{GHz}$ and $f_{2}=10.2\mathrm{GHz}$, respectively. The signal bandwidths are B=100MHz, Δf=0.3125MHz and the number of frequency-hopping points is N=320. The modulated signal from radar 1 $c_{1}(n)$ is a non-repeated random integer sequence of length N in the interval $\left[0,N-1\right]$. The modulated rule of radar 2 can be expressed as $c_{2}(n)=-c_{1}(n)$. The azimuth angle θ and the pitch angle β of the line of sight of the radar in the ship body coordinates system are 30° and 10°, respectively. The initial ranges of radars 1 and 2 are 12 km and 12.02 km, respectively. Suppose that the speed of the radar platform has been compensated by GPS-assisted data. The target velocity is $v_{tLOS}=-4m/s$. The accumulated number of pulses of two-dimensional imaging is 960. The corresponding azimuth synthetic aperture is 1.72.

**Figure 10:**
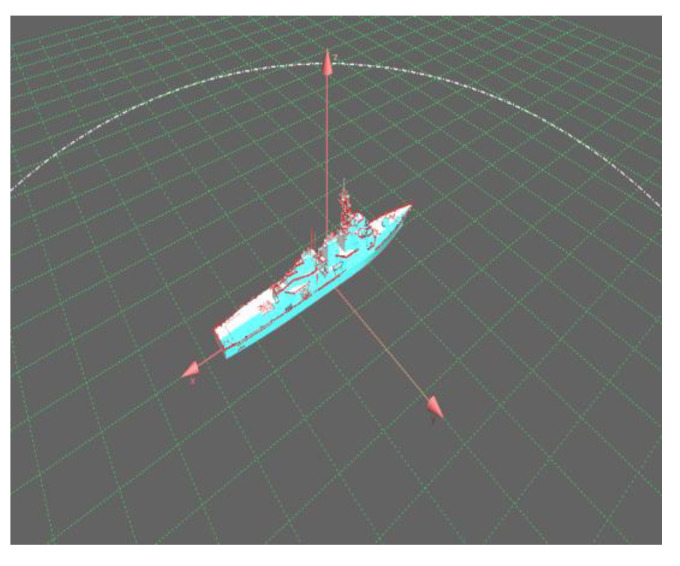
Model of ship.

**Figure 11:**
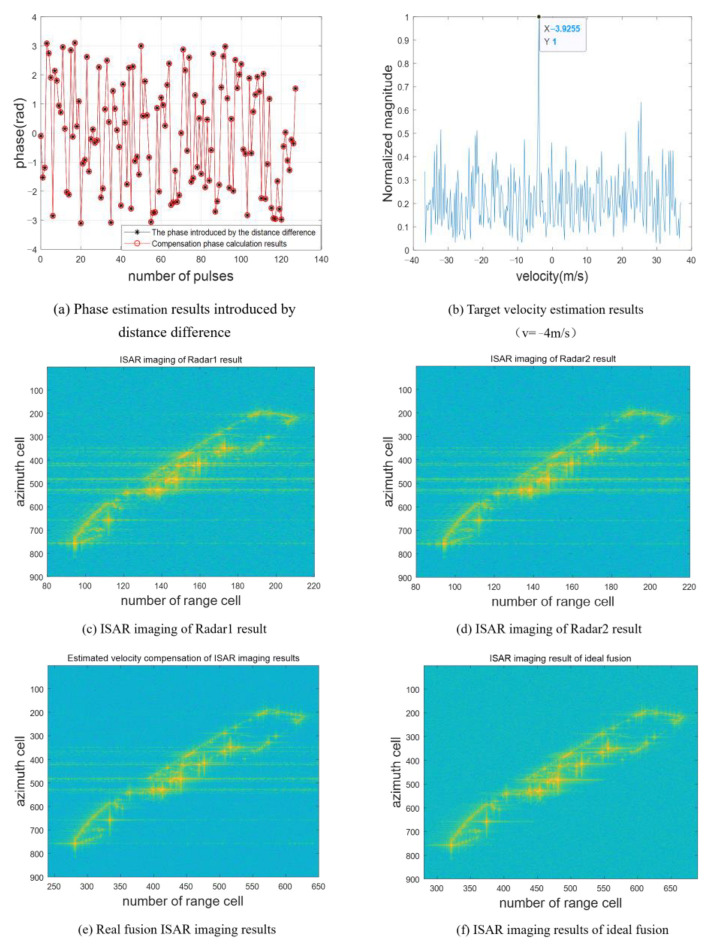
Comparison of imaging results.

**Figure 12:**
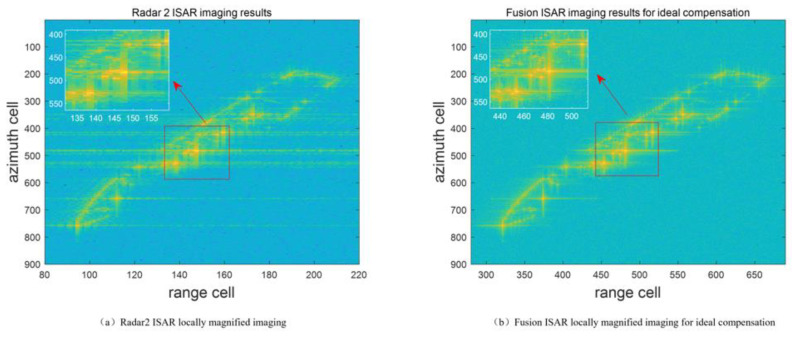
Locally magnified contrast images.

Figure 10 is the model of ship. Figure 11 is comparison of imaging results. Figure 11a,b show the estimated results of the phase introduced by the range difference and the target velocity, respectively. The target velocity estimated from the peak position is −3.925 m/s. It meets the requirements of Equation (15). Figure 11c and d show the independent imaging results of radars 1 and 2, respectively. Due to the limitation of range resolution, the target is distributed around 120 range units in the range direction. The sidelobe of the scattering center has a great influence, resulting in a long trailing. The results obtained by the fusion imaging mode are shown in Figure 11e. The target is distributed around 360 range units in the range direction. With the increase in the number of effective coherent frequency points, the sidelobe of the target scattering center decreases significantly, and the contour becomes clearer. Figure 12a shows the local magnification of the radar 2 ISAR imaging, and Figure 12b shows the local magnification of the fusion ISAR imaging after ideal compensation. By comparing the local magnification area of the two images, the image with ideal compensation has a better focusing effect of scattering center than that of the single base radar imaging. Compared with the ISAR imaging results under ideal velocity compensation (Figure 11f), the estimated contrast of the fused ISAR image after velocity compensation is 104.8591, while the contrast of the ideal compensation ISAR image is 106.4266. The proposed method can realize bistatic cooperative imaging provides high-quality target information for target recognition.

## 6. Conclusions

Bistatic radar cooperation can effectively improve the detection performance of target characteristics. In this paper, a signal-level cooperative imaging method based on random complementary waveforms was studied based on the two-transmitter and two-receiver cooperative detection system of the bistatic radar. Firstly, the echo signals of the two radars satisfy the complementary characteristics and tight constraints are designed. The echo signal model is derived. The compensation phase between echoes is accurately calculated by using the echoes of the homologous transmitted signals. The coherent compensation of the echoes is completed. Furthermore, an efficient velocity estimation method is designed based on the complementary characteristics of the frequency modulation. The estimated result of the target velocity is within the range of velocity accuracy. Finally, a band fusion algorithm based on tight constraint rearrangement and zero complement is used to realize two-dimensional imaging of the bistatic radar with high-resolution. The point-target simulation data and high-frequency electromagnetic calculation data are used to verify the effectiveness of the proposed method. By comparing with the imaging results of the single base radar, the proposed imaging algorithm can provide more target details. The proposed method is expected to provide beneficial exploration and basic algorithm support for the cooperative imaging of missile-borne, dual-based radars.

## Data Availability

The data is unavailable due to privacy restrictions.
